# Acute effects of ultra-short aerobic and resistance, yoga-based mobility, and mindfulness meditation sessions on mental health: a randomized online study

**DOI:** 10.3389/fspor.2026.1774292

**Published:** 2026-06-30

**Authors:** Verena Marschin, Cornelia Herbert

**Affiliations:** Department Applied Emotion and Motivation Psychology, Institute of Psychology and Education, Ulm University, Ulm, Germany

**Keywords:** affect, anxiety, exercise, mindfulness meditation, physical activity, stress, ultra-short activity, yoga

## Abstract

**Introduction:**

Ultra-short activity bouts (<10 min) may represent scalable, low-barrier strategies for improving mental health, an increasingly important goal due to rising stress levels and the need for preventive approaches. Both physical activity (PA) and mindfulness-based practices are associated with psychological benefits but are assumed to operate through partly distinct mechanisms (e.g., physiological activation and affective stimulation in movement-based activities vs. attentional and emotional regulation in mindfulness practices). However, direct comparisons between these modalities in ultra-short formats remain scarce. This randomized online study therefore examined the acute effects of three brief activities—endurance and strength PA (ESPA), yoga-based mobility PA (YMPA), and mindfulness meditation (MM)—on perceived stress, positive and negative affect, and anxiety during the COVID-19 lockdown.

**Methods:**

German adults from the general population and university students (*N* = 131; 18–60 years) were randomized to ESPA (*n* = 40), YMPA (*n* = 45), or MM (*n* = 46). Participants completed a 7-min guided activity, preceded and followed by assessments of perceived stress (PSQ), positive and negative affect (PANAS), and state anxiety (STAI). Linear mixed-effects models examined group, time, and Group × Time effects while adjusting for age, gender, leisure-time physical activity, and student status.

**Results:**

Across conditions, significant improvements from pre- to post-intervention were observed for perceived stress, negative affect and state anxiety. However, no significant Group × Time interactions were detected, indicating that modalities did not differ in their acute effects. The interaction for positive affect was marginally significant (*p* = .05). Positive affect increased most strongly following ESPA, whereas smaller changes were observed in YMPA and almost no change occurred in MM. Baseline distress levels were elevated, consistent with the pandemic context.

**Discussion:**

Findings suggest that even a single ultra-short online activity session can produce immediate improvements in key mental health indicators. Comparable benefits may reflect shared regulatory mechanisms linking physical activity and mindfulness practices. Movement-based activities may additionally enhance positive affect.

**Conclusion:**

Ultra-brief online PA and mindfulness sessions appear similarly effective for short-term improvements in stress, anxiety, and negative affect. Such scalable micro-interventions may inform mental health promotion strategies in everyday settings.

## 1 Introduction

Despite increasing awareness and efforts, improving mental health remains one of the most pressing global health challenges (1). According to the latest WHO data, more than one billion people worldwide live with mental health conditions, and the majority have insufficient access to care (2). Psychological states such as perceived stress, affective well-being, and anxiety represent highly prevalent contributors to quality of life and years lived with disability (3, 4). Importantly, these states are modifiable and can be targeted through preventive lifestyle-based approaches, including regular physical activity (PA), mind-body activities, and mindfulness practices, which are increasingly recognized as protective and accessible strategies to enhance psychological well-being (5, 6).

A growing body of evidence indicates that physical activity can influence psychological states not only in long-term interventions but also following a single exercise bout: Meta-analytic evidence indicates that acute exercise is associated with immediate improvements in affective states, including enhanced mood and reductions in anxiety, across predominantly healthy adult samples [e.g., (7)]. A systematic review focusing specifically on state anxiety also reported small but consistent anxiolytic effects following single exercise bouts (8). Beyond subjective outcomes, acute exercise has also been shown to attenuate stress reactivity across physiological and psychological systems, suggesting rapid regulatory effects on stress-response mechanisms (9). Together, these findings support the notion that even single bouts of PA may serve as an accessible strategy for the immediate regulation of affective and stress-related states. However, existing reviews also highlight substantial heterogeneity in effect sizes and intervention characteristics, reflecting large variations in exercise modality, duration, intensity and study design (7, 8). As a result, controlled comparative studies are needed to reduce heterogeneity and clarify modality-specific effects under standardized conditions.

Importantly, while the number of PA bouts performed is relevant, accumulating evidence suggests that psychological benefits do not necessarily require long durations of activity (10, 11). Meta-analytic findings indicate that even small increases in PA are associated with improvements in anxiety and psychological distress across healthy and clinical adult populations (10, 11). Extending these observations, recent research has increasingly focused on ultra-short exercise bouts, often described as “exercise snacks” or micro-bouts, defined as short, structured activity episodes dispersed throughout the day (12). Earlier terminology occasionally referred to such activities as “non-bout” exercise to distinguish them from previously recommended ≥10-min activity bouts [e.g., (13)]; however, these activities still constitute discrete bouts of PA and are conceptualized here as ultra-short exercise bouts. These ultra-short formats aim to reduce common barriers such as limited time and motivation while promoting adherence and higher compliance in everyday contexts (14–16). Evidence suggests that even ultra-short bouts of light-intensity PA can enhance perceived well-being in healthy young adults under experimental conditions (17). In addition, accelerometer-assessed observational data indicated that the accumulation of moderate-to-vigorous physical activity (MVPA) in episodes lasting <10 min is associated with higher health-related quality of life in adult populations (13). Consistent with these findings, recent PA guidelines no longer require activity to be accumulated in ≥10-min bouts, acknowledging that shorter activity episodes can produce comparable health benefits, provided that the recommended total volume of 150 min of moderate-to-vigorous PA per week is achieved (18, 19). Despite these promising developments, most studies have examined single exercise modalities in isolation and have focused primarily on physiological or workplace outcomes rather than broader psychological states [e.g., (20)].

Alongside PA interventions, mindfulness-based practices have emerged as another accessible strategy for regulating affective and stress-related states (21). Mindfulness meditation (MM) involves the intentional, nonjudgmental awareness of present-moment experiences (22). It has been associated with reductions in stress, anxiety, and negative affect, as well as improvements in psychological well-being across diverse healthy populations (21, 23, 24). Results were also found in university students, with decreased distress (subjectively but also measured as decreased cortisol level) and lower subjective state anxiety, even in comparison to active control groups (25, 26). Similarly, meta-analytic evidence indicates significant small-to-moderate effects of digital mindfulness interventions on depression, well-being, anxiety, and stress (27), highlighting the potential of online delivery to increase accessibility and reduce structural barriers to participation. Importantly, as with PA interventions [e.g., (13, 19)], evidence suggests that even very short mindfulness practices may already be sufficient to elicit psychological benefits: For example, ultra-short mindfulness sessions of approximately 5 min have been shown to produce effects comparable to, or even greater than, longer sessions of 20 min with respect to trait mindfulness, stress, and anxiety (28).

Although both brief physical activity and mindfulness interventions have demonstrated beneficial effects on stress-related and affective outcomes [e.g., (10, 11, 13, 21, 24)], they are typically investigated in isolation. Direct comparisons between different exercise modalities and mindfulness-based practices are scarce, particularly in ultra-short formats (29). The review by Edwards and Loprinzi (29) reported largely comparable effects of exercise and non-active meditation on perceived stress and well-being. Experimental studies examining brief interventions, such as brisk walking and meditation, demonstrated improvements in mood, whereas effects on state anxiety appeared modality-specific (30–32). These findings indicate that modality-specific differences may emerge depending on the outcome assessed. Different exercise modalities may generally engage partially distinct physiological and psychological mechanisms, suggesting that acute responses may vary depending on activity type (10, 30, 31). Endurance and strength-based exercises primarily differ in metabolic demand and autonomic activation, whereas yoga and mindfulness-based practices additionally emphasize attentional regulation and interoceptive awareness (6, 22). Despite these conceptual differences, both physical activity and mindfulness-based practices may also share acute regulatory mechanisms, including attentional shifting, or temporary distraction from stressors and the environment (29–32). Accordingly, comparative designs including multiple exercise modalities alongside mindfulness practices have been explicitly recommended, for example contrasting non-active meditation with mindfulness-based exercise (e.g., yoga-based movement/mobility practices) and non-mindfulness-based exercise [e.g., endurance or strength training; (29)]. Yet, such designs remain largely absent from the empirical literature, although they may help clarify modality-specific effectiveness as well as potential underlying mechanisms (10, 30, 31). This is essential for identifying efficient and scalable strategies that can be implemented in time-constrained real-world settings.

Previous work from our research group has examined short-term effects of brief multimodal activity breaks in healthy adult and student populations, demonstrating acute changes in affective and cognitive outcomes (33–36). However, these studies did not directly contrast distinct exercise modalities with mindfulness practices within a standardized ultra-brief online framework. From an applied perspective, digital delivery formats may represent a particularly suitable context for brief mental health interventions, as they allow flexible implementation within daily routines while reducing logistical barriers to participation (37). Online interventions have demonstrated beneficial effects on psychological well-being and may enhance accessibility and adherence compared to traditional in-person approaches (21, 28, 38). Importantly, digitally delivered activities are typically performed in participants’ natural environments, increasing ecological validity but also introducing variability in implementation conditions (39). Despite their growing relevance, to the best of our knowledge, no studies to date have directly compared different PA modalities with mindfulness meditation within fully online ultra-brief intervention settings, leaving their relative effectiveness under real-world conditions largely unknown.

Notably, many prior brief intervention studies have been conducted primarily in university student samples [e.g., (25, 26, 38)], which may limit generalizability to broader adult populations (21). The present study therefore included both students and non-student adults recruited from the wider community. Examining brief interventions within a more heterogeneous adult sample and digitally delivered format may enhance ecological validity and applicability across diverse real-world contexts.

Taken together, the present study aimed to directly compare the acute effects of three ultra-brief activity modalities delivered in an online setting: (1) endurance and strength exercise, (2) yoga-based mobility, and (3) mindfulness meditation. Primary outcomes were pre-to-post changes in perceived stress, positive and negative affect, and state anxiety. Research on low-to-moderate-intensity ultra-brief PA remains underrepresented (7), highlighting the importance of such comparisons. Based on theoretical considerations suggesting both shared and partially distinct physiological and psychological mechanisms across modalities (6, 22), as well as previous findings indicating generally beneficial, but also outcome-specific effects (29–31), we hypothesized that all three interventions would lead to acute improvements in psychological outcomes from pre- to post-intervention. However, we further expected these improvements to differ in magnitude and potentially in their underlying mechanisms depending on the modality, resulting in modality-specific patterns across outcomes. Given the currently limited and heterogenous comparative evidence, these differential effects were explored in a hypothesis-driven but partly exploratory manner. In addition, we examined overall pre-to-post changes across outcomes irrespective of intervention modality.

## 2 Materials and methods

This online study was part of a bigger research project of the Department Applied Emotion and Motivation Psychology at Ulm University. The current data and quantitative analysis are based on single acute ultra-short (<10 min) sessions of different activities to improve mental health. This online study was planned and carried out during the first wave of COVID-19. It was originally planned as laboratory study to monitor physical activity and the whole experimental process, which was not possible to carry out due to COVID restrictions.

### 2.1 Participants

Recruitment for the study took place via flyer distribution, oral presentation and the university-intern e-mail distribution lists from the commencement of April to the midpoint of May 2020 in the south of Germany. As compensation, participants were entered into a raffle to win online shopping vouchers. Psychology undergraduates could alternatively choose to get course credits for their study curriculum. Participation was voluntary and written informed consent was provided prior to participation. Ethical approval was obtained by the local medical ethics commission of Ulm University Germany (https://www.uni-ulm.de/einrichtungen/ethikkommission-der-universitaet-ulm/, 56/20).

University students as well as other people of every age above 18 years and a high proficiency of the German language were included. Participants were excluded from study participation if they had any injuries or physical illness where physical activity could pose a health risk. This was measured via the Physical Activity Readiness Questionnaire [PAR-Q; (40)]; translated by Dept. Applied Emotion and Motivation Psychology, Ulm University). The item about dizziness or loss of consciousness was left out because these symptoms do not constitute an exclusion criterion for our activities. In addition, participants were excluded if they were under 18 years of age. Screening for inclusion and exclusion criteria took place right at the beginning of the online questionnaire after informed consent via the online survey tool LimeSurvey Professional (Vers. 6; https://www.limesurvey.org).

### 2.2 Measures

#### 2.2.1 Subjective physical activity

The German version of the Global Physical Activity Questionnaire [GPAQ; (41, 42)] was utilized to assess physical activity levels and sedentary time. The GPAQ, developed by the World Health Organization (42), is a comprehensive tool designed to measure PA overall and in three subdomains: work, travel to and from places, and leisure physical activities. Participants are asked to recall their usual physical activity within one week, providing insights into the frequency (number of days), intensity (i.e., specification in moderate and vigorous intensity) and duration in hours and minutes of their activities. Participants can be categorized as low, moderately, or highly active based on established GPAQ classification criteria: low activity (<600 MET-min/week), moderate activity (≥600 MET-min/week or equivalent combinations of moderate and vigorous activity), and high activity [≥3,000 MET-min/week or ≥1,500 MET-min/week of vigorous activity; (42)]. MET values quantify the relative energy expenditure of an activity compared to resting metabolic rate (123). Accordingly, MET-minutes integrate both the intensity and duration of physical activity into a standardized metric (43). For context, current World Health Organization guidelines correspond to approximately 600 MET-minutes per week (43). On top of that, sedentary time per day in hours and minutes is requested in the GPAQ (41, 42). Reliability ranged from *Kappa* = .67 to .73 (*Spearman's rho:* 0.67–0.81). Test-retest reliability ranged from 0.83 to 0.96 within a time span of 10 days or from 0.53 to 0.83 after three months (44). In some studies, the GPAQ also showed concurrent and criterion validity, although this needs to be examined further (45, 46).

#### 2.2.2 Perceived stress

Perceived stress was measured via the short version of the Perceived Stress Questionnaire [PSQ; German modified version; (47)]. The original version by Levenstein et al. (48) was adapted, translated into German and validated by Fliege et al. (47). The full version consists of 30 items, while the short version encompasses 20 items (PSQ-20). Items are rated on a four-point Likert-type scale ranging from 1 = *almost never* to 4 = *usually*. It measures perceived stress burden during the last four weeks. The instructions were slightly adapted to refer to the current experience of stress in order to be able to measure acute intervention effects from pre- to post-measurement. Although not usually done, this approach is theoretically consistent with conceptualization of perceived stress as a dynamic and situational construct (49). The PSQ-20 consists of four scales: Worries, tension, joy and demands. For this study, the total score was used as overall perceived stress level. During the analysis, the mean value of all items is calculated, which is then transformed into values ranging from 0 to 100. Higher scores depict a higher perceived stress level. We refrain from addressing the normal reliability and validity of the questionnaire, since the instructions were changed. Internal consistency was high (.94 and .95) for both measurement points, suggesting that the adapted instructions did not compromise reliability. On top of that, the correlation between pre- and post-scores (*r* = .87) indicated high temporal stability, supporting the measure's reliability while reflecting expected changes across the measurement.

#### 2.2.3 Positive and negative affect

The German version of the Positive and Negative Affect Schedule (PANAS) was used to assess positive and negative affect of participants [German version by (50)]. Originally developed by Watson et al. (51), the PANAS comprises two 10-item affect-related adjectives designed to measure both positive and negative affect. Participants are asked to rate the extent to which they experienced each of the 20 emotions (e.g., interested, distressed, excited, upset) at this moment on a five-point Likert scale ranging from 1 = *not at all* to 5 = *extremely*. The PANAS is well-validated and widely used in psychological research, offering high reliability (*Cronbach's alpha* = .86 for positive and negative affect) and content and construct validity. For analysis, the mean score of 10 items was calculated once for positive and once for negative affect. Cronbach's alpha for positive affect was 0.91, for negative affect it was 0.85 (50).

#### 2.2.4 Anxiety

The State-Trait Anxiety Inventory [STAI; (52)] is a self-report assessment tool which was employed to assess anxiety levels among participants, distinguishing between state anxiety (temporary condition) and trait anxiety (general condition). State anxiety was used as pre-post-measurement and is therefore of main interest. The STAI was originally developed by Spielberger et al. (53) and was later translated into German by Laux et al. (52). It consists of 20-items each (for state and trait anxiety). Participants rate each item on a four-point Likert scale, ranging from 1 = *not at all* to 4 = *very much so* for state anxiety, and from 1 = *almost never* to 4 = *almost always* for trait anxiety. The answers of the trait scale employed in this study were later excluded because wrong answer options were given (the same were used as for the state scale), hence the validity would be questionable. The STAI has been extensively validated, possessing convergent and criterion validity, and is recognized for its reliability in measuring anxiety across diverse populations and settings [Cronbach's alpha ranging from .90 to .94 for the state scale (52),]. Sum scores of the state scale are used for analysis.

### 2.3 Procedure

After completing the online screening procedure and confirming eligibility, participants accessed the study via an online survey link. All study procedures were completed remotely using the LimeSurvey platform (https://www.limesurvey.org). Participants were automatically randomized to one of the intervention conditions via the built-in randomization function of LimeSurvey at the very beginning. Following randomization, participants completed demographic questions (sex, age, country of origin, marital status, and employment), as well as baseline assessments including mood and arousal ratings and respective pre-intervention outcome measures. In the middle of the questionnaire, an embedded 7-min video or audio, corresponding to the randomized condition, was played. Participants were instructed to complete the guided exercises. During video and audio playback, progression within the survey was technically blocked to ensure that the exercise was viewed continuously before proceeding. The intervention was conducted remotely due to COVID-19 restrictions; participants completed the exercise independently in their own environment, without supervision. Immediately after completion of the exercises, participants completed the post-intervention assessment consisting of the same outcome measures administered at baseline. Thus, post-measurements were obtained directly following the intervention within the same session. Compliance was assessed via self-report at the end of the survey, where participants indicated whether they had carried out the exercise as instructed. Participants were additionally asked whether they would consider using similar exercises in the future to support their mental health and, if not, to provide reasons in an open-text response format.

#### 2.3.1 Ultra-short intervention conditions

This one short session randomized controlled experiment had three arms: one group with endurance and strength exercises (ESPA), one group with yoga-based and mobility PA (YMPA), and one group with mindfulness meditation (MM). The mindfulness meditation group was purposefully chosen as an active control condition, as it is expected to influence mental health while not involving PA, thereby allowing us to control for non-specific intervention effects.

According to the original study protocol, a fourth activity condition was planned that included the same exercises as the yoga-based and mobility PA group but with an additional focus on breathing. During a *post-hoc* quality control of the intervention materials, we re-examined the video recordings used in the experimental sessions. Upon retrospective review, we identified indications that, in a subset of sessions, the video assigned to the fourth condition (yoga with breathing focus) may not have differed from the standard yoga condition. Because this review was conducted after data collection, it was not possible to verify with absolute certainty whether the intended breathing-focused video was consistently administered. Consequently, condition fidelity for these 53 participants could not be unequivocally established. To preserve internal validity and avoid potential misclassification bias, these cases were conservatively excluded from the final analyses.

All exercises as well as the meditation were carefully chosen and reviewed by two certified fitness and yoga experts, as well as an independent sports scientist. The exercises are detailed in Table 1.

**Table 1:** Description of exercises for different ultra-brief activitie*s.*

Activity group	Exercises	Duration	Components of physical fitness
Endurance and strength	Calf raises	60 s	Muscular endurance
	Front kicks	60 s	Cardiovascular and muscular endurance
	Criss cross jumps	60 s	Cardiovascular endurance
	Forward lunge	60 s	Muscular endurance and strength
	Torso side bend right	60 s	Muscular endurance and strength
	Torso side bend left	60 s	Muscular endurance and strength
	Forward arm circles	60 s	Muscular endurance
Yoga and mobility	Body awareness	60 s	Body awareness and mindfulness
	Upward salute (“Urdhva Hastasana”)	60 s	Balance, strength and flexibility
	Upright standing yoga mudra	45 s	Flexibility, mobility and strength
	Backward shoulder rolls	45 s	Flexibility and mobility
	Forward shoulder rolls	45 s	Flexibility and mobility
	Head rotation	45 s	Flexibility and mobility
	Head tilt right and left	45 s	Flexibility and mobility
	Head tilt forward and backward	45 s	Flexibility and mobility
	Body awareness	30 s	Body awareness and mindfulness
Mindfulness meditation	Breathing meditation	7 min	Mindfulness

Components of physical fitness were discussed with an independent sports scientist. The depicted categorization here is based on the American College of Sports Medicine (55). Yoga poses are derived from Iyengar (54).

ESPA primarily comprised exercises targeting cardiovascular and muscular endurance and strength. The audio accompanying the video emphasized correct exercise execution, with occasional motivational messages such as “Well done.” The classification of exercise components was developed in consultation with an independent sports scientist and aligned with established components of physical fitness as defined by the American College of Sports Medicine (55). The selected exercises primarily consisted of functional bodyweight movements performed continuously for 60 seconds each within a brief circuit format. Several exercises (e.g., lunges, front kicks, and criss-cross jumps) simultaneously elicited cardiovascular activation and muscular endurance demands. Given this integrated physiological stimulus and the short duration of the intervention, a strict distinction between endurance and strength modalities was not considered conceptually meaningful. Therefore, endurance- and strength-oriented exercises were combined into a single combined endurance–strength condition representing general physical activation.

The YMPA video included low-intensity yoga-inspired, as well as flexibility and mobility exercises, emphasizing joint mobility and gentle activation. At the beginning and end of the video, participants were guided to direct their attention toward bodily sensations, in order to promote body awareness. Participants were instructed to briefly notice physical sensations without evaluation and to observe changes in bodily perception.

The audio guidance for MM centered on mindfulness regarding one's breathing. The full text can be viewed under S1 (translated to English for this publication; the original German text can be obtained from the authors).

Exercises were guided by a female person each, with the same female voice-over for all activities. Physical exercises were performed in a standing position without any equipment, whereas MM was practiced while being in a seated position. The duration of activities was 7 min.

ESPA and YMPA exercises were presented via recorded videos, the meditation was presented as audio file. A written introduction preceding the video or audio screen instructed participants to follow the exercises demonstrated in the video (for physical activity exercises) or the oral instructions provided in the audio (for mindfulness meditation). A countdown timer prevented participants from advancing until the designated time elapsed.

#### 2.3.2 Manipulation check

At the end of the questionnaire, participants were asked whether they had completed the assigned activity as instructed. As adherence was assessed via self-report, this procedure cannot fully exclude inaccurate reporting; however, survey progression was technically restricted during intervention playback to increase compliance. Following an intention-to-treat approach, all randomized participants were retained in the analyses regardless of self-reported adherence.

### 2.4 Data analyses

An *a priori* power analysis was conducted using G*Power 3.1 (56, 57). Assuming a medium effect size [*f²* = 0.15; effect size is based on findings of PA and meditation interventions on mental health, i.e., (11, 58)], *α* = .05, and statistical power of .80, the required total sample size for the planned between-group comparisons was estimated at *N* = 103. In addition, a simulation-based sensitivity analysis was conducted for the planned linear mixed-effects model following recommendations for mixed-effects designs by Brysbaert and Stevens (59). Assuming a standardized Group × Time interaction effect of *d* = 0.40, a pre–post correlation of *ρ* = 0.60 [typical for psychological repeated-measures outcomes; (59, 60)], and 5% missingness at post-assessment, approximately 65 participants per group were required to achieve 80% power.

Data analysis was carried out in RStudio, version 2026.01.0. To examine potential baseline differences between the three intervention groups, several statistical tests were conducted depending on the variable type. For continuous variables (age and leisure-time PA), one-way analyses of variance (ANOVA) were performed with group as independent variable. When appropriate, pairwise group comparisons were conducted using estimated marginal means (EMMs) with Holm correction for multiple testing. Effect sizes were calculated as partial eta squared (*η²_p_*). If the assumption of homogeneity of variances was violated, Welch's ANOVA and corresponding Welch-adjusted pairwise *t*-tests with Holm correction were additionally computed. For pairwise comparisons, Cohen's *d* was calculated as effect size estimate. For categorical variables (gender: male/female and student status: student/non-student), chi-square tests of independence were conducted. Effect sizes were calculated using Cramer's *V*. Intervention effects on mental health outcomes were analyzed using linear mixed-effects models (LMMs). Separate models were estimated for all primary outcomes. Models included group (ESPA, YMPA, MM), time (pre vs. post), and the Group × Time interaction as fixed effects, with the interaction representing the primary test of differential intervention effects across the three modalities. Age, gender, leisure-time physical activity, and student status were included as covariates due to their known prognostic influence on mental health. Participant-specific random intercepts were included to account for the repeated-measures structure of the data. Models were estimated using restricted maximum likelihood (REML), and significance tests were obtained using Type III tests with Satterthwaite approximations of the degrees of freedom. Where appropriate, pairwise comparisons based on estimated marginal means were conducted with Holm correction for multiple testing. Statistical significance was set at *p* < .05 (two-tailed). The analyses were performed as intention-to-treat analysis and were conducted using linear mixed-effects models including all available repeated outcome data from randomized participants. Linear mixed-effects models were selected as the primary analytic approach because they use all available repeated outcome data and provide valid estimates under the missing-at-random assumption without requiring explicit imputation. This approach is consistent with methodological work suggesting that, in most situations, linear mixed-model analyses without imputation are appropriate for intention-to-treat analyses in randomized trials with missing follow-up data, and they yield results comparable to multiple imputation approaches (61–65). Due to this, no imputation was used in the present analysis.

As a sensitivity analysis complementing the mixed-effects models, autoregressive ANCOVA models were conducted for each psychological outcome. Post-intervention scores were regressed on the corresponding baseline score, group, sex, age, recreational PA, and student status. Adjusted group means were estimated using estimated marginal means, and pairwise group comparisons were tested using Holm-adjusted contrasts.

## 3 Results

### 3.1 Sample characteristics

**Figure 1:**
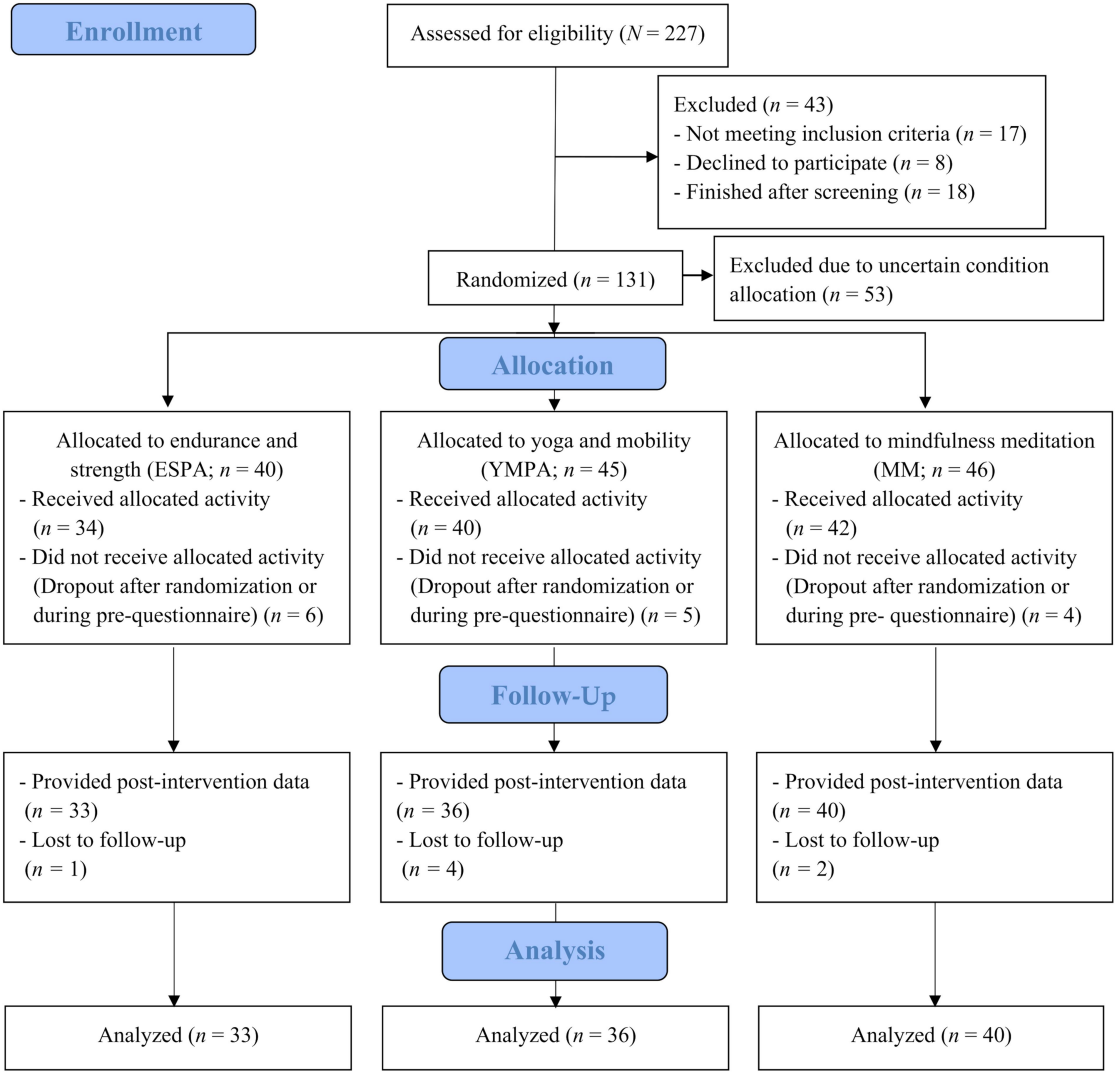
CONSORT flowchart for study samples, drop-outs and exclusions. PA, physical activity. Modified from Hopewell et al. (66). Licensed under Creative Commons Attribution 4.0 International (CC BY 4.0 license, https://creativecommons.org/licenses/by/4.0/).

Figure 1 depicts the process from recruitment to final analysis concerning sample sizes as well as the randomization process. Of all randomized participants, 16.79% dropped out. The highest attrition occurred immediately after randomization or during the pre-measurement phase. The final sample for analysis consisted of *n* = 40 in the combined endurance and strength condition, *n* = 45 in the yoga-based and mobility PA condition, and *n* = 46 in the mindfulness meditation condition.

Mean age of participants was 28.39 years (*SD* = 10.56), ranging from 18 to 60 years. The overall sample consisted of 39 male (29.80%) and 85 female participants (64.90%), *n* = 2 participants reported to be diverse (1.53%) and *n* = 5 didn’t report their sex (3.82%). The sample included *n* = 62 (47.33%) students. Among the non-student participants, the largest proportion worked in social and education professions (22.03%), followed by commercial and administrative occupations (16.95%). Health professionals (10.17%) and technical or engineering workers (10.17%) were equally represented. Smaller proportions were employed in IT and media fields (8.47%), public service or security roles (5.08%), and sports or coaching (3.39%). Remaining occupations that could not be clearly assigned to predefined occupational sectors were grouped into an “other” category (23.73%; e.g., unemployed, household roles, general employment or managerial positions).

### 3.2 Baseline and prognostic variables

Mean physical activity time per week was 709.73 min (*SD* = 632.51 min; *n* = 115). This equates on average 3,682.57 MET (metabolic equivalent of task) minutes per week (*SD* = 3,250.37). *N* = 9 participants were categorized as low (7.83%), *n* = 55 (47.80%) as moderately active, and *n* = 51 (44.40%) as highly active. Mean leisure physical activity time per week was 333.37 min (*SD* = 307.65 min). Participants were sitting on average for 505.96 min per day (*SD* = 223.10 min), which equals 8.43 h per day.

In accordance with the CONSORT 2010 statement (67) and recommendations by de Boer et al. (68) and Harvey (69), no formal significance tests should be conducted for baseline group differences after randomization. Nonetheless, for reasons of completeness, distribution differences were examined: No significant differences were found between groups concerning student status, *x*^2^(2) = 2.53, *p* = .283, Cramer's V = 0.14, or leisure-time PA, Welch's *F*(2,72.74) = 0.37, *p* = .695. A Welch's ANOVA revealed significant differences in age between groups, Welch's *F*(2,79.48) = 5.20, *p* < .01. *Post-hoc* pairwise comparisons showed that ESPA had a significantly higher age than YMPA, *p* < .01 (Holm corrected), *d* = 0.71, *CI*_95_ = [0.26, 1.16], and than MM, *p* < .05 (Holm corrected), *d* = 0.55, *CI*_95_ = [0.11, 0.99]. The proportion of women and men differed between groups, *x*^2^(2) = 6.22, *p* < .05, Cramer's V = .22. Participants identifying as diverse or “other” were excluded from inferential testing of baseline differences due to low cell frequencies.

Although some baseline differences in age and gender were observed, such imbalances are expected by chance in randomized designs (68, 69). More importantly, both age and gender are well-established prognostic variables in mental-health research (70) and, consistent with current methodological recommendations (68), were included in all models not only for adjustment of baseline group differences but also for enhanced precision of treatment effect estimates and internal validity (71). In addition, leisure-time PA was included as further prognostic variable.

### 3.3 Descriptive statistics

Descriptive statistics for the outcome variables are presented in Table 2.

**Table 2:** Means, standard deviations and reliability of mental health variables at baseline and at follow-u*p.*

Outcome	Time	ESPA (*n* = 40)	YMPA (*n* = 45)	MM (*n* = 46)	Total (*N_Pre_* = 116) (*N_Post_* = 109)	*α*
PSQ perceived stress (%)	Pre	36.81 (19.46)	41.04 (19.30)	30.63 (21.73)	36.03 (20.56)	0.93
	Post	29.34 (19.97)	29.68 (19.28)	26.33 (20.83)	28.35 (19.94)	0.94
PANAS positive affect (1–5)	Pre	2.89 (0.85)	2.96 (0.82)	3.04 (0.82)	2.97 (0.83)	0.91
	Post	3.22 (0.85)	3.25 (0.78)	2.97 (0.84)	3.14 (0.82)	0.92
PANAS negative affect (1–5)	Pre	1.54 (0.59)	1.41 (0.39)	1.42 (0.51)	1.45 (0.50)	0.84
	Post	1.40 (0.53)	1.20 (0.26)	1.27 (0.52)	1.28 (0.46)	0.88
STAI state state anxiety (20–80)	Pre	40.03 (11.43)	39.10 (10.11)	36.07 (12.23)	38.28 (11.33)	0.93
	Post	37.48 (11.63)	33.77 (7.67)	33.38 (11.01)	34.78 (10.32)	0.93

ESPA, endurance and strength physical activity group; YMPA, yoga-based and mobility physical activity group; MM: Mindfulness meditation group. PSQ, Perceived Stress Questionnaire [German modified version; (47)], higher scores depict higher stress; PANAS, Positive and Negative Affect Schedule [German version by (50)], positive: higher scores depict higher positive affect; negative: higher scores depict higher negative affect; STAI, State and Trait Anxiety Inventory (52), higher scores depict higher anxiety.

Overall perceived stress [PSQ (47)] at baseline was higher in all groups compared to norm values (72). Positive affect was lower than norms in all groups (50). Negative affect at baseline was lower than norm values (50). Generally, all affect scores correspond to typical values reported for non-clinical samples (73). For the STAI state (52) norm values for a German sample are lacking, because it is mostly used as change measure. Baseline values of state anxiety were close to a suggested cut-point of 39–40 as clinically significant [e.g., (74)].

### 3.4 Intervention effects

Table 2 shows that all groups exhibited improved mental health following the activities compared to their pre-states. Perceived stress as well as state anxiety temporarily dropped from clinically significant levels to more normative levels (47, 74). The only exception was positive affect, which did not change much in the MM group, but in both PA groups.

The first hypothesis stated that all intervention groups would show improvements in perceived stress, state anxiety, and positive and negative affect from pre- to post-intervention. In addition, it was hypothesized that these changes would differ between groups, reflecting modality-specific effects. Linear mixed-effects models with random intercepts for participants were conducted to examine the effects of group, time, and their interaction on psychological outcomes while controlling for sex, age, leisure-time PA, and student status. Results are presented in Table 3.

**Table 3:** Mixed-model effects of ESPA, YMPA and MM on primary outcome*s.*

Outcome	Effect	*F*(df1,df2)	*p*	*η²_p_*
PSQ perceived stress	Group	0.83 (2,108.27)	.438	.02
	Time	58.41 (1,106.01)	<.001	.36
	Group × Time	3.05 (2,106.01)	.051	.05
PANAS positive affect	Group	0.45 (2,106.69)	.639	.01
	Time	9.07 (1,104.24)	.003	.08
	Group × Time	3.08 (2,104.25)	.050	.06
PANAS negative affect	Group	1.34 (2,108.61)	.266	.02
	Time	27.50 (1,106.39)	<.001	.21
	Group × Time	0.17 (2,106.40)	.842	<.01
STAI state state anxiety	Group	0.99 (2,107.55)	.374	.02
	Time	31.37 (1,103.61)	<.001	.23
	Group × Time	0.67 (2,103.61)	.513	.01

ESPA, endurance and strength physical activity group; YMPA, yoga-based and mobility physical activity group; MM: mindfulness meditation group; PSQ, Perceived Stress Questionnaire [German modified version; (47)], PANAS, Positive and Negative Affect Schedule [German version by (50)], STAI, State and Trait Anxiety Inventory (52).

Across outcomes, no significant Group × Time interactions were observed. However, the interaction for perceived stress approached statistical significance, *p* = .051. It has to be noted, that this effect was not supported in additional autoregressive supplementary ANCOVA models controlling for baseline values (see Supplementary Material S2). The interaction effect for positive affect was also marginally significant in the mixed model, *p* = .050. In contrast to perceived stress, the supplementary autoregressive ANCOVA model yielded a largely similar result for positive affect, with a significant effect for group (see Supplementary Material S2). Significant main effects of time indicated overall improvements for perceived stress, positive affect, negative affect, and state anxiety from baseline to post-exercise. No significant main effects of group were found in the mixed models.

## 4 Discussion

The present study examined the acute effects of three ultra-short online interventions—endurance/strength exercise, yoga-based mobility PA, and mindfulness meditation—on perceived stress, affect, and state anxiety, and whether these effects differ between intervention modalities. The main finding was that all three interventions were followed by improvements in perceived stress, negative affect, and state anxiety, whereas no clear differential effects between modalities emerged in the mixed-model analyses. Thus, our primary hypothesis of modality-specific acute effects was not supported for these variables.

While our hypothesis was based on theoretical considerations suggesting partially distinct physiological and psychological mechanisms across modalities, it also acknowledged that these interventions share common regulatory pathways (75). Specifically, ESPA, YMPA and MM may all induce acute psychological benefits through mechanisms such as attentional distraction, shifts in affective processing, or short-term physiological regulation (29–32). Against this background, we expected overall improvements across all conditions, but tentatively hypothesized that modality-specific patterns might emerge depending on the outcome. However, the absence of differential effects in the present study suggests that, under ultra-short intervention conditions, these shared mechanisms may dominate over modality-specific processes. This interpretation is consistent with previous empirical findings indicating broadly comparable benefits of both physical activity and mindfulness-based interventions. Meta-analytic evidence indicates that even a single bout of exercise can enhance mood and reduce anxiety in healthy adults (7, 8). Similarly, mindfulness interventions have repeatedly been associated with reductions in stress and anxiety across diverse populations (21, 23, 24). Direct comparisons between exercise and meditation are relatively scarce, particularly in ultra-short formats, limiting the ability to derive clear modality-specific predictions, but available evidence suggests broadly comparable benefits across modalities. For example, randomized trials comparing exercise-based and mindfulness-based interventions reported significant improvements in stress and affect in both conditions without consistent superiority of either approach (75, 76). Likewise, a review by Edwards and Loprinzi (29) concluded that exercise and meditation can produce comparable improvements in perceived stress and well-being. Taken together, these findings suggest that although modality-specific mechanisms may differ, ultra-short interventions across modalities may converge in their immediate psychological effects.

Acute studies have reported mixed findings for specific outcomes such as state anxiety. Edwards and Loprinzi (29–31) observed mood improvements following both brisk walking and meditation, whereas reductions in state anxiety were primarily found after meditation in Edwards et al. (32). However, such differences did not emerge in the present study, which may be related to differences in intervention characteristics. Differences in exercise modality may partly explain these inconsistencies, as brisk walking differs substantially from the exercises used in the present ESPA condition. In addition, many yoga-based interventions combine movement, breathing, and mindfulness components, making it difficult to isolate the relative contribution of physical vs. attentional processes (77, 78). By contrast, the present study directly contrasted three conceptually distinct modalities within the same standardized design, allowing a clearer comparison of their pure acute psychological effects. The present findings extend previous work by showing that very brief online activities of less than 10 min may already produce measurable short-term psychological benefits, at least for stress- and anxiety-related outcomes. Most previous exercise and mindfulness studies have used sessions lasting approximately 20–60 min (7, 8, 21, 23, 24).

Mechanisms underlying the observed improvements in mental health for all conditions may be interpreted within the framework of the neurovisceral integration model (79, 80). This model emphasizes the functional integration of brain systems involved in cognitive, affective, and autonomic regulation, particularly within the central autonomic network (CAN), linking prefrontal and limbic structures with autonomic nervous system activity (81–83). More efficient regulation within this network has been associated with improved emotional regulation and stress resilience (80, 82–85). Taken together, there might be overlapping regulatory processes for both PA and MM, linking brain and autonomic function. These processes may potentially increase interoceptive awareness of bodily states (75), although this was not directly assessed in the present study. Such shared mechanisms may help explain the comparable short-term improvements observed across modalities. Future research should examine these potential mechanisms more directly, for example by including measures of interoceptive awareness.

While theoretical models such as the neurovisceral integration framework suggest that MM may enhance prefrontal regulatory control over limbic and autonomic processes, thereby supporting emotion regulation and reductions in stress and anxiety (83, 84, 86–89), physical activity may influence similar regulatory systems through partially different pathways, including neurochemical changes, improved autonomic balance, and enhanced resilience of stress-related physiological systems (90–96). However, these modality-specific mechanisms may require longer duration, greater intensity, or repeated exposure to become detectable. In the present study, the absence of differential effects may indicate that under ultra-short intervention conditions, shared regulatory processes dominate over these more specific pathways. Future studies combining psychological outcomes with physiological measures, such as electrocardiography or impedance cardiography, may help to disentangle common and modality-specific mechanisms.

Above all, data collection took place during the first wave of the COVID-19 pandemic, a period characterized by elevated psychological distress and anxiety across many populations (97–103). Consistent with this context, baseline levels of perceived stress and state anxiety in the present sample were relatively elevated and close to clinically relevant thresholds. Such heightened baseline distress may have increased the potential for short-term improvements following brief regulatory activities. Consequently, the magnitude of pre–post changes observed in this study may partly reflect the broader psychosocial circumstances during the pandemic and the potential responsiveness to brief digital interventions (70, 104), which may limit generalizability of findings.

A nuanced pattern of findings in the present study emerged for positive affect. In the primary mixed-model analyses, the Group × Time interaction did not reach conventional statistical significance, although the effect approached the threshold of significance. A similar trend was observed for perceived stress, but this effect was not robust across analytical approaches and may be influenced by baseline differences between groups, as indicated by the autoregressive models controlling for initial values (also see Supplemental Material S2); it is therefore not further interpreted. Inspection of the observed means indicated small increases in positive affect in the ESPA condition, whereas smaller changes were observed in the YMPA group and no clear increase occurred in the MM group. Supplementary autoregressive ANCOVA models indicated a significant group effect for positive affect, with higher adjusted post-intervention values in the endurance and strength group compared with the mindfulness meditation group. Because this pattern was not consistently supported across analytical approaches, the finding should be interpreted cautiously and no firm conclusions can be drawn. The differences between statistical findings might generally reflect limited statistical power to detect small-to-moderate Group × Time interaction effects in the mixed-effects models due to a limited number of participants, whereas power for autoregressive models was sufficient, as also indicated by the *a priori* power analysis. This difference in analytical approach may partly explain why the interaction effect for positive affect did not reach statistical significance in the mixed models.

Although these findings should be interpreted cautiously, the observed pattern may still provide insight into potential modality-specific mechanisms. One possible explanation is that movement-based activities may produce stronger immediate increases in positive affect due to physiological activation and energizing effects associated with physical exertion, which has also been found in previous research (105). At the same time, the absence of a clear increase in the mindfulness condition contrasts with some studies reporting affective benefits following brief mindfulness practice [e.g., (30, 106, 107)]. However, affective outcomes were operationalized differently in several of these studies, often including dimensions such as revitalization or tranquility rather than positive affect *per se* (30), which may partly explain the discrepancy. On top of that, the interventions were delivered fully online in participants’ natural environments, thereby reflecting real-world digital health applications and the increasing use of digitally delivered mental health interventions (28, 38, 39). This context may also be relevant when interpreting the affective responses observed in the present study.

### 4.1 Limitations, strengths and implications for future research

Several limitations should be considered when interpreting the present findings. Firstly, self-report measures may be subject to biases (108), e.g., inaccurate recalls or faking good or bad, just to name a few. However, they remain widely used and well-established tools for assessing mental health, specifically in online settings (109). In our case, the instructions of the PSQ [German modified version; (47)] were slightly changed to capture perceived stress as a state measure. This was not validated, therefore results concerning perceived stress have to be interpreted with caution. Psychometric analysis supports the use in the current dataset, but this does of course not replace a formal validation. Concerning the physical activity assessment via the GPAQ (41, 42), an overestimation is possible, and the categorization of activities to moderate- or vigorous-intensity might also be biased (110, 111). An overestimation in our data is likely, as relatively few participants were classified in the low-activity group. A better option would be to assess PA via wearable monitors [e.g., accelerometry or pedometers; (112)]. Alternatively, the relatively high physical activity levels observed in the sample might also suggest that many participants were already meeting or exceeding recommended guidelines. This may have influenced the observed effects, as individuals with higher baseline activity levels may respond differently to acute interventions compared to less active populations (113). This pattern may have been further amplified by the COVID-19 pandemic and the online recruitment strategy, which may have preferentially attracted individuals with a stronger interest in health management behaviors such as physical activity, stress reduction, or mindfulness practices, thereby limiting representativeness and generalizability.

Secondly, the study was originally planned as a laboratory-based experiment but had to be conducted remotely due to COVID-19 restrictions during the data collection period. Consequently, adherence to the intervention could not be objectively verified, and it cannot be fully guaranteed that all participants performed the activities exactly as instructed, despite the use of a self-reported manipulation check. Although such self-report measures cannot entirely exclude inaccurate reporting, survey progression was technically restricted during intervention playback, increasing the likelihood that participants were at least exposed to the intervention as intended. At the same time, conducting the intervention in participants’ natural environments increased ecological validity and aligns with the aim of informing scalable interventions that can be performed independently and in self-selected settings. Moreover, many individuals were already accustomed to home-based exercise formats, a trend further accelerated during the COVID-19 pandemic (114).

The type of delivery medium (video vs. audio) may also have influenced the results. Video-based instructions may be more engaging because participants can visually mirror a real person rather than relying solely on auditory cues and visual and auditory information can be processed simultaneously, which can facilitate behavioral engagement (115). Future studies should therefore systematically control for differences between media formats.

Due to the behavioral nature of the interventions, participants could not be blinded to the type of exercise performed. This may have introduced expectancy effects, particularly given the reliance on self-reported mental health outcomes. However, allocation was concealed prior to assignment through automated randomization, and the inclusion of an active control condition (mindfulness meditation) may have reduced differential expectancy effects between groups.

One potential limitation concerns the timing of the psychological assessment directly following PA: Direct measurement after PA may partly reflect physiological arousal or activation rather than real changes in affective state, or state anxiety. To minimize these potential confounding effects, the state anxiety questionnaire was administered at the end of the post-measurement, allowing participants’ heart rate and arousal levels to return closer to baseline before assessing their emotional state. Nevertheless, most accurately, future research should consider including additional post-exercise measurement points (e.g., after 10 or 20 min). While simple measures such as heart rate can capture general arousal, more detailed psychophysiological assessments (e.g., electrocardiography or impedance cardiography) would allow a more precise examination of autonomic regulation, which may be more closely linked to affective and stress-related processes (83, 86).

Some might argue that the lack of a passive control group limits the ability to make direct comparisons to a non-active state. However, researchers state that in a lot of cases, active control groups are the better choice, since they are better comparable in terms of expectancies, motivation, or cognitive strains, and there is evidence that they work equally well as passive control groups in terms of no higher risk for inflation or bias (116). On top of that, passive control groups are more prone to expectancy effects, demand characteristics or placebo effect (116).

Although randomization was not stratified by gender or age and some baseline imbalances occurred, these differences are expected by chance in randomized designs. In line with the CONSORT 2010 statement and recommendations by de Boer et al. (68), inferential tests for baseline differences should not be overinterpreted. Nevertheless, age and gender, as well as student status and leisure-time PA were included as covariates in all models to statistically adjust for potential prognostic effects, thereby improving precision and internal validity of the estimated intervention effects. Missing post-intervention data were not imputed. Instead, primary analyses relied on linear mixed-effects models using all available repeated outcome data, which provide valid estimates under the missing-at-random assumption and are considered an appropriate approach in randomized trials (61–65). However, if missing data were not at random, this may have influenced the estimates.

Although student status was included as a binary covariate in all models to account for potential differences between university students and non-student adults, the study was not designed to examine subgroup-specific intervention effects. Future research should investigate whether the effectiveness of ultra-short mental health interventions differs across these populations.

Notwithstanding these limitations, there are several strengths that should be highlighted. The most important is the direct comparison of endurance and strength, yoga-based mobility PA, and mindfulness meditation. To the best of our knowledge, these three different activities have never been directly compared before, although taken for themselves, they already have a good scientific foundation (117). All activities were chosen and shown by experts in their fields, ensuring a high level of standardization and content validity. Finally, the use of ultra-short interventions represents a novel and practically relevant approach, as such brief activities may be more easily integrated into daily life and chances are higher that participants will carry out the activities more often and adhere to them over a longer time period.

Although both PA and MM improved mental health outcomes, a growing body of research suggests that combined interventions may offer additive or synergistic benefits [e.g., (6, 76, 118–120)]. The lack of strong group differences in the present study suggests that hybrid interventions may capitalize on shared regulatory mechanisms while potentially enhancing affective outcomes. Future research should therefore directly compare isolated and combined modalities, particularly within ultra-short protocols. In addition, future studies should examine longer-term effects, include follow-up assessments, and investigate more diverse populations, including older adults and individuals with mental or physical health conditions (121, 122). To improve generalizability and practical relevance, more cross-cultural studies are warranted to help tailor mental health strategies to diverse but also specific populations, which further enhances public health impact and accessibility.

## 5 Conclusion

The study advances the field by showing that even a single ultra-short (< 10 min) bout of activity can lead to acute measurable mental health/psychological benefits. Different modalities with or without physical activity were equally effective for reducing perceived stress, anxiety, and improving negative affect. Positive affect enhancement could be more specific to physically active, more arousing brief exercises—a nuance not well-documented in prior short-duration studies. The results highlight the potential of brief, accessible online interventions for adult populations with promising avenues for longer mental health and physical activity interventions.

## Data Availability

The original contributions presented in the study are included in the article/Supplementary Material, further inquiries can be directed to the corresponding authors.
