# Supplementary material for: Acute effects of ultra-short aerobic and resistance, yoga-based mobility, and mindfulness meditation sessions on mental health: a randomized online study
Source: Front Sports Act Living. 2026 Jun 30;8:1774292. doi: 10.3389/fspor.2026.1774292 (PMC13366608; doi:10.3389/fspor.2026.1774292)
Supplement: Supplementary file 1 [file Supplementaryfile1.docx]

**Supplemental materials**

**S1**

*Instructions for mindfulness meditation (translated from German by DeepL)*

*„The mindful sitting exercise with breath observation. The classic way to practice concentration and calm the mind by focusing on the breath.*

*Take your time to find a comfortable sitting position. Free, not leaning, and upright.*

*Your hands can rest on your lap, your eyes closed.*

*Notice the contact with the surface you are sitting on, your buttocks, feet, your body as a whole.*

*Then direct your attention to the movements of your breath, in your chest, in your abdomen and from there to the sensations that your breath causes in your nose.*

*The subtle sensations of the flow of breath at the nostrils, perhaps minimal stretching and relaxation.*

*And you may also notice the flow of air at the upper lip.*

*Choose one aspect from these areas that you can perceive well. A point on which you can rest your attention. And once you have chosen a point, just stay there.*

*Just observe.*

*The in and out.*

*Just let the focus of your perception rest there.*

*And if you notice that your mind starts to wander on its own, just keep returning to the chosen point of observation.*

*Return again and again. And leave everything as it is.*

*You don't need to change anything.*

*Just observe.*

*In and out.*

*And when you notice that your mind has wandered, just return.*

*Return to this in and out.*

*Just observe. And stop.*

*If you notice that your mind has wandered off, simply bring it back to your breath.*

*And now, please get ready to return your attention to the outside world.*

*Perhaps with a few deep breaths.*

*With a stretch and a yawn.*

*And then open your eyes again.“*

**S2**

*Supplementary results for autoregressive ANCOVA models*

| **Outcome** | ***N*** | **Baseline predictor** | **Group effect** | ***Partial η²*** | **Significant pairwise comparisons** |
| --- | --- | --- | --- | --- | --- |
| *PSQ Perceived stress* | 108 | *F*(1,100) = 360.81,  *p* < .001 | *F*(2,100) = 1.59, *p* = .209 | .03 | None |
| *PANAS Positive Affect* | 107 | *F*(1,99) = 134.66, *p* < .001 | *F*(2,99) = 3.40,  *p* = .037 | .06 | ESPA > MM,  Δ = 0.34,  95% *CI* [0.02, 0.67], *p* = .037 |
| *PANAS Negative Affect* | 107 | *F*(1,99) = 133.95, *p* < .001 | *F*(2,99) = 0.71, *p* = .492 | .01 | None |
| *STAI (state)* | 106 | *F*(1,98) = 256.46,  *p* < .001 | *F*(2,98) = 0.96,  *p* = .387 | .02 | None |

*Note.* Autoregressive ANCOVA models predicted each post-intervention outcome from its respective baseline value, group, gender, age, leisure-time physical activity, and student status. ESPA = Endurance and strength physical activity group; MM = Mindfulness Meditation; PSQ = Perceived Stress Questionnaire (German modified version**;** Fliege et al., 2009); PANAS = Positive and Negative Affect Schedule (German version by Breyer, and Bluemke, 2016); STAI = State and Trait Anxiety Inventory (Laux et al., 1981). Pairwise group comparisons were Holm-adjusted. Partial η² values refer to the group effect.

As a sensitivity analysis complementing the mixed models, autoregressive ANCOVA models were fitted for post-intervention perceived stress, positive affect, negative affect, and state anxiety, controlling for the respective baseline score, group, gender, age, leisure-time physical activity, and student status. In all models, the baseline score significantly predicted the post-intervention outcome (all ps < .001). A significant group effect emerged only for positive affect, F(2, 99) = 3.40, p = .037, partial η² = .06. Holm-adjusted pairwise comparisons indicated that the endurance and strength group showed higher adjusted positive affect than the mindfulness meditation group, mean difference = 0.34, *95% CI* [0.02, 0.67], p = .037, d = 0.65. No significant group effects were observed for perceived stress, negative affect, or state anxiety (all ps ≥ .209). These findings were broadly consistent with the mixed-model results, which showed overall improvements over time and only limited evidence for differential group effects.
